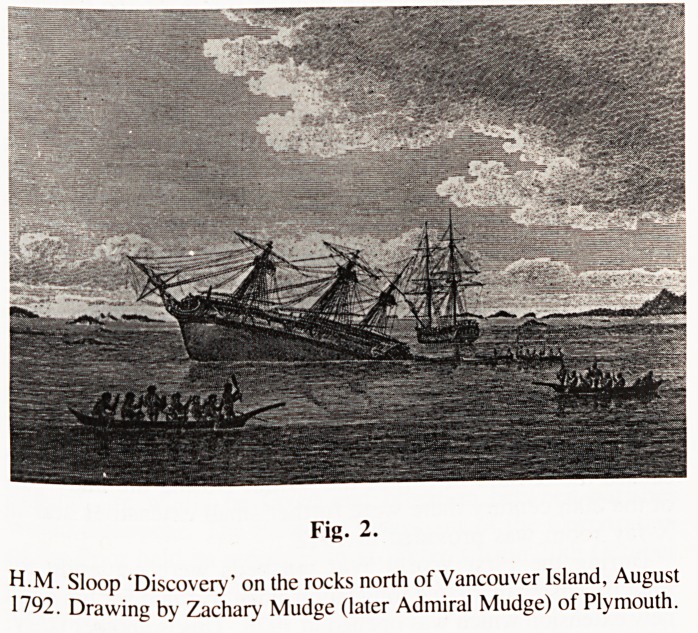# Archibald Menzies, Surgeon Botanist

**Published:** 1991-12

**Authors:** John Naish


### Archibald Menzies: Surgeon Botanist

**John Naish, MD, FRCP**

**Fig. 1.**

Archibald Menzies, MD, FLS, 1754-1852.
Oil painting by A. Upton Ellis hanging in the R.Linnean Society building, Piccadilly, London.

Archibald Menzies, the 2nd son of a Highland gardener, was born in 1754. His father was the head gardener at Castle Menzies near Loch Tay, and the clan leader's distant relative. From him Archibald learnt respect for authority and the joy of growing things. In an atmosphere of secure prosperity young Archibald had a good practical and general education, working in the Castle gardens and grounds from an early age.

At the age of 14, he went to Edinburgh as a garden apprentice to Professor John Hope who was re-establishing the Royal Botanic Garden on a new site on Leith Walk. Hope was a physician and a botanical disciple of Linnaeus, Hope's genius for diplomacy as well as science enabled him to coax sufficient money from the Crown to re-site the Royal Botanic Garden and a Regius Chair of Botany for himself. This he was able to combine with a physician's practice and, later, with the Chair of Medicine at the University and, for a time, the Presidency of the Royal College of Physicians, Hope's qualified students went out into a widening world to serve as Surgeons at sea, with the East India Company or to the Americas. Many became devoted plant collectors, providing their revered professor and the Botanic Gardens with packets of seeds and roots from most parts of the world.

Archibald Menzies longed to be a plant collector, too, so he was encouraged to qualify as a surgeon, during which time he spent one youthful summer plant-collecting in the Highlands for two prominent London physicians, Drs. Fothergill and Pitcairn. They paid his expenses and published his work.

Menzies only practised for less than a year as a civilian surgeon — in Caernarfon of all places; no doubt the botany of Snowdonia was the motive for chosing such a remote and tiny place. Then, in 1781, he joined the R.N. as surgeon and was soon seeing active service with Rodney's Fleet in the Caribbean. He was present at the Battle of the Saintes in April 1782 which effectively ended the long-drawn-out war with the U.S.A.

In the peacetime Navy from 1783 onwards Menzies was fortunate to continue in permanent employment and to come into correspondence with Sir Joseph Banks, to whom he sent packets of seeds and plant material from Nova Scotia and Labrador, Sir Joseph was a great catch. He was a personal friend of King George, he was rich, President of the Royal Society, and a confidential adviser to Ministers of the Crown. Banks thought highly of the young Highlander's accuracy, dependability and draughtsmanship. The geniality and enthusiasm of the young man also shine through his letters.

In 1786, through Banks' influence, he obtained permission from the Admiralty to join a commercial voyage of exploration to the West Coast of America under the command of Lieut. James Colnett, R.N. He had two tiny ships, the *Prince of Wales* and the *Princess of Wales,* fitted out at the expense of a wealthy London merchant who hoped that the transport of Sea Otter furs from Alaska to China would reap a nice 500% profit. Cook's men, on his last voyage, had discovered the high value which the Chinese put on the soft luxuriant fur of the Sea Otter. After the posthumous publication of his journal in 1784 there was a rush to fit out ships from this trans-pacific trade, and this led to commercial clashes between Russian, Spanish, British and American adventurers.

During Colnett's circumnavigation of the world Menzies witnessed the ravages of scurvy, but he quickly learnt which common weeds could be served as anti-scorbutic greens to his men and how to brew spruce beer. Nootka on the west coast of what was later to be called Vancouver Island was the gathering place for the fur traders, and there Menzies made many valuable botanical discoveries as well as learning how to find suitable green food and berries. It was his subsequent proud boast at the end of the voyage that he had only lost one man, and he died from an infection contracted in China. Menzies missed the foundation of the Linnean Society in London, but he was soon elected a Fellow after his return in 1789. The founder of the Society, Sir. J. E. Smith, named a genus of the heather family — found by Menzies in Alaska — as *Menziesia.*

The greatest adventure of Archibald's life began in 1791 when he went as botanist on a Naval voyage of exploration into the Pacific. There were political and commercial reasons behind this venture which was masterminded by Sir J. Banks. The Commander was a young man of 35, George Vancouver, the grandson of a Dutch immigrant to Kings Lynn. He was a man of unreliable temper, who nevertheless achieved great accuracy in his survey of the North-West coast of America from California to Alaska which took three whole summers to complete, the winters being spent in California and Hawaii.

Menzies kept a journal, and sent regular letters to Banks, nearly all of which survive. His journal has never been published but it is a marvellous record of adventure, natural history and anthropology. I have been able to read all of it on microfilms provided by the British Museum and the National Library of Australia.

For Menzies it was a heaven sent opportunity to explore the natural history of many unknown regions, and the large numbers of trees, shrubs, garden plants, mosses, ferns and lichens which now bear his name are a perpetual testimony to his pioneer efforts. He was the first to describe the botany of S.W. Australia, the first to describe the unique mosses and ferns of Dusky Sound, S.New Zealand and the sub-alpine plants of Hawaii. In America, he described the Arbutus which he called the Oriental Strawberry Tree but which is now known as A. menziesii, The Douglas Fir, the Sitka Spruce and both the parents of the ever popular hybrid the Leyland Cypress. His accurate ethnological observation of the Coastal Indians whose stone-age, non-ceramic culture was embellished by superb woodwork, carving, basket-weaving and pictorial art, and whose skill in mime, rhetoric and song was such a source of wonder to the young sailors from the West are now recognised as a precious anthropological heritage. In the Hawaiian Islands, which Vancouver put under the protection of King George to save them from the brutal exploitation of the fur-traders and the privateers, Menzies was — long after — remembered as "the red-faced man who cut off the legs of man and gathered grass". This genial Scot was an understanding friend to the Polynesians, as he was to the West Coast Indians whose habit of drenching their bodies in fish oil and painting themselves with ochre normally set up an olfactory barrier to close acquaintance.

He was thge first man, ever, to climb the 13500 foot mountain of Mauna Loa in Hawaii, taking six days of hard travel to reach the snow line. He estimated its height with his portable barometer and his estimate proved to be only 50′ out. His thoughts when he lay shivering through the night on a bare rock below the summit are, in my view, quite enchanting. His journal is a treasure trove of accurate observation and percipient feeling.

Adventures there were in plenty, some dangerous, some uncomfortable, some amusing. One of the most memorable was when he and Puget climbed to the top of a rock in British Columbia to explore an abandoned Indian village, the stench was unbearable, and before long they were faced with something worse, Hundreds of fleas ". . . fixed themselves to our shoes stocking and cloths in such incredible number that the whole party was obliged to quit the rock in great precipitation." As soon as they had scrambled down the ladder and got to the water some stripped naked and plunged in the sea, immersing their clothes at the same time, but all to no avail, for their enemies afterwards ". . . leapt about as frisky as ever." Even towing their clothes behind the boats didn't kill them so, that night, they had to boil the lot.

**Fig. 2.**

H.M. Sloop 'Discovery' on the rocks north of Vancouver Island, August 1792. Drawing by Zachary Mudge (later Admiral Mudge) of Plymouth.

*H.M.S. Discovery* in which Menzies sailed had a garden frame — designed by Banks — on the Quarterdeck, so that he could bring back live plants for H.M. Garden at Kew. It never worked, Dogs, Cats and Pigeons kept creeping in when the plants were being ventilated; tarry drippings from the rigging poisoned the soil and spring temperatures of −12°C in Alaska killed off all his Hawaiian plants. It was a constant source of friction between Vancouver and Menzies which ended in a blazing row on the quarterdeck in July 1795 — just as the voyage was coming to an end, Menzies arrived home under arrest for standing up to Vancouver's tongue-lashing in defence of his precious plants.

Somehow, the quarrel was patched up without a court martial and Menzies spent the next few years working on his material in Banks' London Library and herbarium, while he enjoyed the sinecure appointment of surgeon to the royal yacht.

In 1798, he was chosen to supervise an experiment to control an outbreak of contagious fever — undoubtedly Typhus — which had broken out in a Russion squadron lying off Sheerness and was threatening the British hospital ships. The idea was to fumigate the hospital ship with Nitric Oxide, then very much in vogue. As well as almost asphyxiating his patients with brown fumes according to orders, Menzies also had all the beds scrubbed and the clothes boiled, so, if the Nitric Oxide didn't kill the lice, at least the washing did. The epidemic came under control, and the method was written up by Sir Carmichael Smyth.

After futher naval service in the Caribbean during which Menzies developed Asthma, he returned to London in 1802 and was invalided out of the Navy at the age of 46. He then married a Scots girl in her late thirties and set up as a surgeon in a house North of Oxford St. In the following year he was made M.D. Honoris Causa of Aberdeen. He did well in practice, but it left him no time to write up his botanical discoveries. His great ambition was to produce a major work on the Cryptogams, since so many of the new mosses, ferns and lichens were of his own discovery. In old age his good memory and prowess as a raconteur made him popular with botanists old and young, especially William Jackson Hooker, the father of the Sir Joseph Hooker. One famous botanist, meeting him when he was still a student, described him "as a truly kind old man" and was amazed by his extraordinary memory for events of long ago.

His death at the age of 88 came swiftly, but he was able to complete and sign his Will leaving his extensive herbarium to the Royal Botanic Gardens of Edinburgh. There you can still see his dried specimens and meticulous descriptions, while in the British Museum of Mankind near Burlington Arcade you can see the precious collection of Indian artefacts which he gave to King George on his return in 1795.

He was a man who made an immense impression on his contemporaries, but is largely forgotten today, though his warm and generous personality endeared him to several succeeding generations of botanists who honoured his name in many plants and trees which are now a much loved part of our landscape. The Douglas Fir has been renamed to commemorate Menzies, *Pseudotsuga menziesii*, but his life and charming character are today almost unknown among educated men; which is why I have presented him to the exceptionally well educated members of the Bristol Medico-Historical Society.

## Figures and Tables

**Fig. 1. f1:**
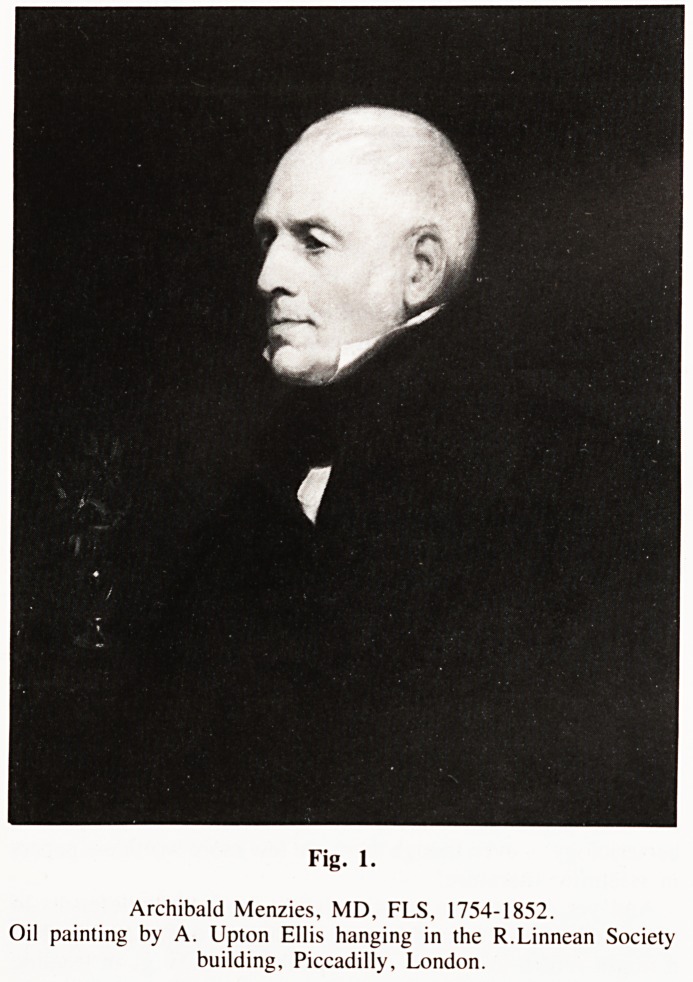


**Fig. 2. f2:**